# Structure–Stability Relationship in Aqueous Colloids of Latex Particles and Gemini Surfactants

**DOI:** 10.1021/acs.jpcb.2c06259

**Published:** 2022-10-26

**Authors:** Dóra Takács, Tamás Péter, Zsófia Vargáné Árok, Bojana Katana, Snežana Papović, Slobodan Gadzuric, Milan Vraneš, István Szilágyi

**Affiliations:** † MTA-SZTE Lendület Biocolloids Research Group, Department of Physical Chemistry and Materials Science, University of Szeged, 6720Szeged, Hungary; ‡ Department of Chemistry, Biochemistry and Environmental Protection, Faculty of Sciences, University of Novi Sad, 21 000Novi Sad, Serbia

## Abstract

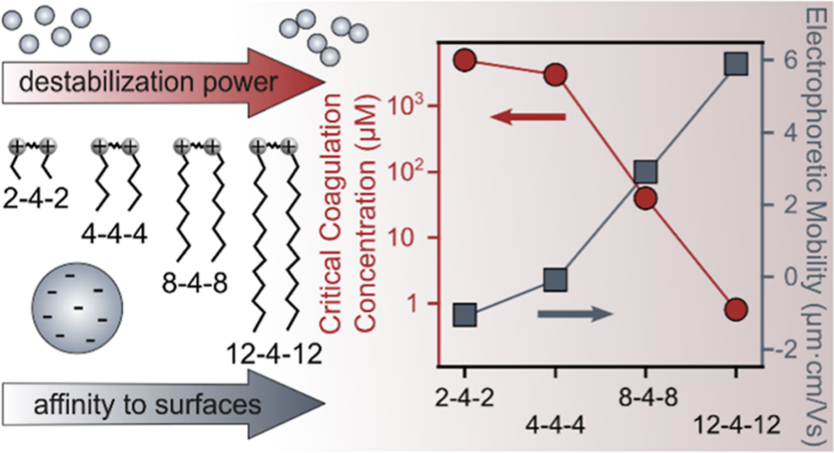

The influence of gemini surfactants (GSs) on the charging and aggregation features of anionic sulfate modified latex (SL) particles was investigated by light scattering techniques in aqueous dispersions. The GSs of short alkyl chains (2-4-2 and 4-4-4) resembled simple inert salts and aggregated the particles by charge screening. The adsorption of GSs of longer alkyl chains (8-4-8, 12-4-12, and 12-6-12) on SL led to charge neutralization and overcharging of the particles, giving rise to destabilization and restabilization of the dispersions, respectively. The comparison of the interfacial behavior of dimeric and the corresponding monomeric surfactants revealed that the former shows a more profound influence on the colloidal stability due to the presence of double positively charged head groups and hydrophobic tails, which is favorable to enhancing both electrostatic and hydrophobic particle–GS and GS–GS interactions at the interface. The different extent of the particle–GS interactions was responsible for the variation of the GS destabilization power, following the 2-4-2 < 4-4-4 < 8-4-8 < 12-4-12 order, while the length of the GS spacer did not affect the adsorption and aggregation processes. The valence of the background salts strongly influenced the stability of the SL-GS dispersions through altering the electrostatic interactions, which was more pronounced for multivalent counterions. These findings indicate that both electrostatic and hydrophobic effects play crucial roles in the adsorption of GSs on oppositely charged particles and in the corresponding aggregation mechanism. The major interparticle forces can be adjusted by changing the structure and concentration of the GSs and inorganic electrolytes present in the systems.

## Introduction

Surface engineering of colloidal or nanoparticles by surfactant adsorption is a commonly applied protocol to control dispersion stability or to provide specific functionalities.^1,2^ These amphiphilic substances are widely used in materials synthesis,^3,4^ water and environmental pollution control,^5^ corrosion inhibition,^6^ and in the petroleum industry.^7^ One of the reasons for their popularity as surface-active agents is that they can significantly alter interfacial properties,^8,9^ and hence, their adsorption from aqueous solutions to surfaces is often used to regulate surface characteristics, for example, to change the wetting and adhesion properties of a solid material.^10−12^ They also have a significant impact on the charging features of dispersed particles, which greatly affect their aggregation behavior and their ultimate bioavailability and toxicity in aqueous environments.^13,14^ As a result, many studies have focused on the qualitative and quantitative aspects of surfactant adsorption on particle surfaces.^2,15−18^

Besides, the interfacial feature of surfactants is a key issue in enhanced oil recovery too, as it affects the efficiency of chemical flooding and thus the economic viability of these projects.^19−21^ Studies have shown that surfactant adsorption is influenced by the temperature, salinity, pH, and other reservoir parameters.^22^ Common examples of these approaches, therefore, include adjusting the ionic content of the injection water^7,23^ or the addition of nanoparticles.^24^ Regarding surface-active compounds applied in oil recovery, the gemini surfactants (GSs) are among the most promising new generation candidates.^25,26^ They contain two hydrophilic head groups and two hydrophobic aliphatic chains linked by a so-called spacer located at or near the head groups.^27^ In recent years, they have attracted considerable attention in both fundamental research^28^ and industrial applications^25,29^ since, when compared to traditional single-chain surfactants, GSs possess various advantageous properties such as enhanced interfacial activity, better water solubility, lower critical micelle concentration, specific aggregation behavior, and interesting rheological properties. These unusual features of GSs are the basis of their applications in nanoparticle synthesis,^30^ enhanced oil recovery,^25^ and corrosion inhibition, for instance.^31^ Previously reported research data showed that the main factors which affect interfacial adsorption behavior are the length of hydrophobic alkyl chains and spacer groups, the polarity of the head group, and the type of counterions.^28^

Although the interaction of GSs with solid surfaces such as clay,^32^ silica,^28,33^ titania,^16,34^ and zinc oxide nanoparticles^35^ has been previously investigated to some extent, only a few studies were undertaken to determine the effect of GSs on the colloidal stability of aqueous particle dispersions. In particular, systematic determination of the surface charge and aggregation rates of nano or colloid particles while varying the experimental conditions (e.g., ionic strength, pH, structure, and concentration of GSs) is missing. Since surfactants and (nano)particles are likely to coexist in aqueous environments and industrial processes, further studies on their effects on particle dispersion stability are crucial, as the colloidal stability greatly influences both the success of the application and the environmental impact.

Therefore, the present study aims for a comprehensive investigation of charging and aggregation of latex particles in the presence of GSs. To scrutinize the effect of the composition of GSs, the influence of the structure (alkyl chain and spacer length were varied) on the colloidal stability was assessed by electrophoretic and time-resolved dynamic light scattering techniques. The interfacial behavior of a GS was compared to the conventional monomeric counterpart to assess the advantages of the dimeric structure. In addition, the stability of latex-GS samples was investigated in different ionic environments by changing the composition and valence of the background electrolyte. The findings will attract considerable attention in both fundamental and applied research since it provides a valuable tool for screening GSs to find the best candidates for desired applications, wherever stable or unstable particle dispersions must be designed.

## Methods

### Materials

Negatively charged sulfate-modified latex (SL) particles were purchased from Thermo Fisher Scientific. The manufacturer determined the particle diameter as 430 nm with a relative polydispersity of 1.8% by a transmission electron microscope, while the reported charge density, obtained by conductometric titration, was −12 mC/m^2^. Inorganic salts, such as potassium chloride (KCl), potassium sulfate (K_2_SO_4_), calcium chloride (CaCl_2_), as well as the dodecyltrimethylammonium bromide (DTAB), were bought from VWR and were used without further purification. Ultrapure water (VWR Puranity TU+) was used. The temperature of the measurements was 25 °C and pH 4 was adjusted with HCl (VWR). All salt stock solutions and water were filtered with a 0.1 μm syringe filter (Millex) to avoid dust contamination.

### Synthesis and Characterization of GSs

**Scheme 1:**
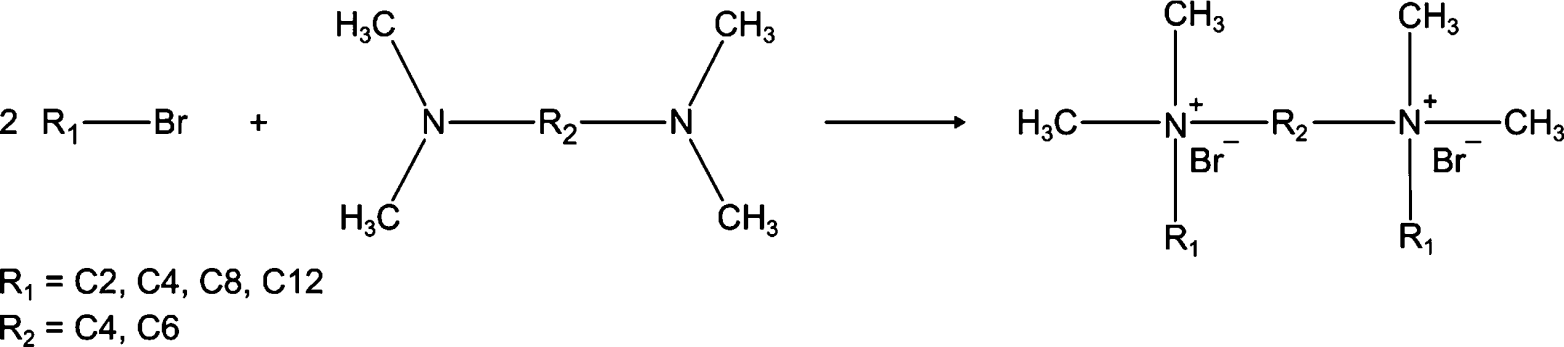
Synthetic Pathway of GSs

The provenance and purity of starting compounds for the synthesis of the GSs are given in Table S1 and were used as received without further purification. The preparation of *N*,*N*′-diethyl-*N*,*N*,*N*′,*N*′-tetramethylbutane-1,4-diammonium dibromide (2-4-2), *N*,*N*′-dibutyl-*N*,*N*,*N*′,*N*′-tetramethylbutane-1,4-diammonium dibromide (4-4-4), *N*,*N*,*N*′,*N*′-tetramethyl-*N*,*N*′-dioctylbutane-1,4-diammonium dibromide (8-4-8), *N*,*N*′-didodecyl-*N*,*N*,*N*′,*N*′-tetramethylbutane-1,4-diammonium dibromide (12-4-12), and *N*,*N*′-didodecyl-*N*,*N*,*N*′,*N*′-tetramethylhexane-1,6-diammonium dibromide (12-6-12) was accomplished by mixing 1 mole of diamino alkanes and 2.2 mol of *n*-alkyl bromides (Scheme 1). Reactants were dissolved in acetonitrile and stirred for 24 h at room temperature, then at 60 and 120 °C under reflux for 2 h and 3 days, respectively. The remaining solvent was removed using a rotary evaporator. Unreacted compounds were eliminated by extraction with ethyl acetate, which was repeated three times. The obtained GSs were dried under vacuum for the next 24 h to remove any traces of the used solvent and stored in a vacuum desiccator over P_2_O_5_ before use.

**Figure 1:**
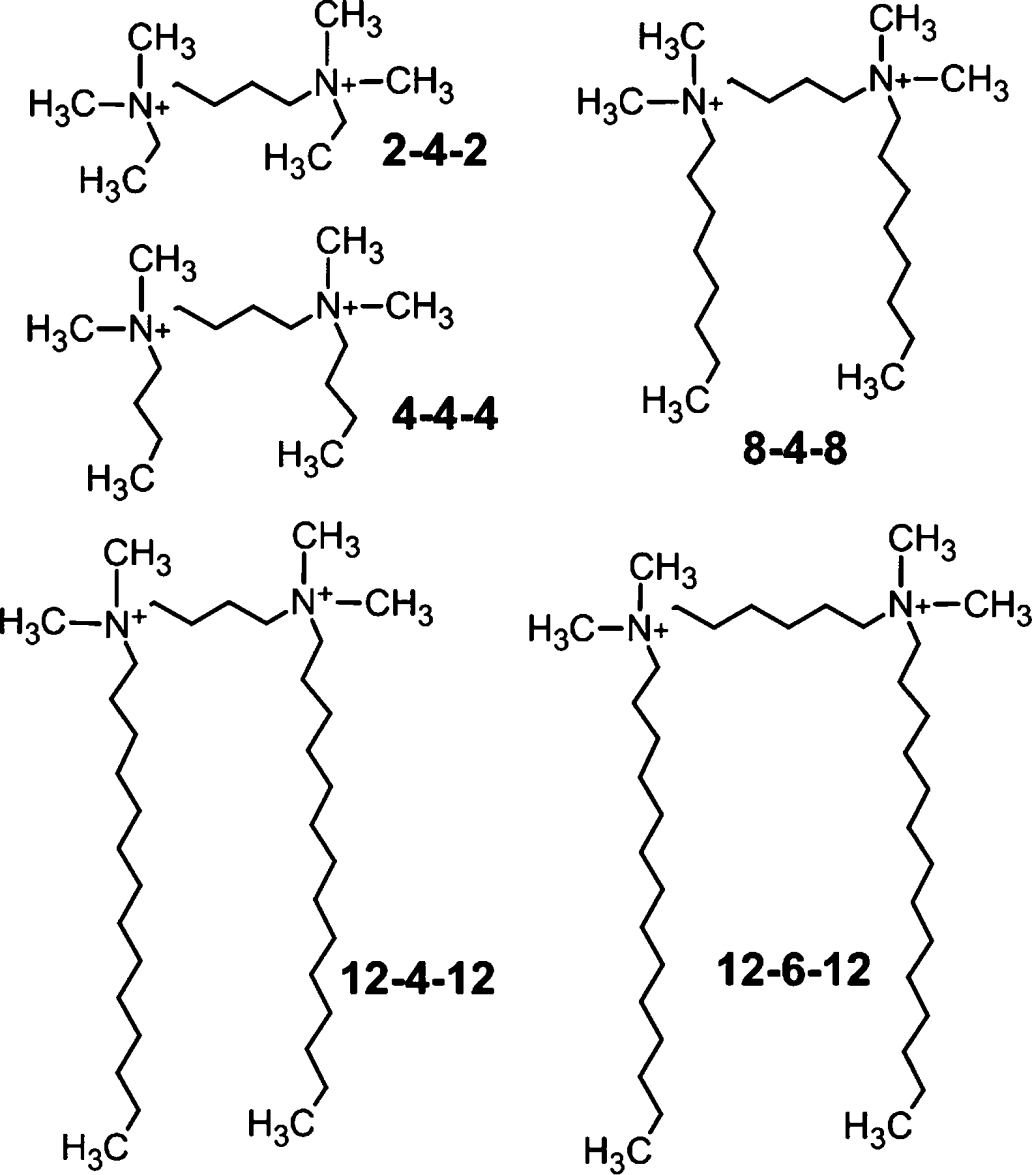
Cation structure of the GSs studied in the present work.

The successful synthesis of the GSs was confirmed by ^1^H and ^13^C NMR analysis. The experimental protocols and the results of these measurements, including the spectra (Figures S1–S5) and peak assignment, are shown in the Supporting Information. The thermal stability of GSs was determined by simultaneous thermogravimetric and differential scanning calorimetry measurements (Table 1 and the methodology together with data analysis are discussed in the Supporting Information). The chemical structures of the GSs are shown in Figure 1.

**Table 1:** Characteristic Data of GSs Used in This Work

symbol	*T*_onset_ (°C)^a^	*k*_fast_(×10^–18^ m^3^/s)^b^	IEP (μM)^c^	CCC (μM)^d^
2-4-2	270.3	2.19		5000
4-4-4	241.1	2.01		3000
8-4-8	225.9	1.44	270	40
12-4-12	221.4	1.01	1.8	0.8
12-6-12	225.3	1.15	1.4	0.5
DTAB		0.69	170	30

a The onset temperature of thermal decomposition.

b Absolute aggregation rate coefficient in the fast aggregation regime obtained by eq 3.

c Concentration of GSs needed to reach the IEP with SL particles.

d Experimentally obtained GS concentrations, which are required to attain the critical coagulation concentration (CCC) with sulfate latex particles.

### Electrophoresis

Electrophoretic mobility was measured using a Litesizer 500 instrument (Anton Paar) with a laser source (658 nm wavelength) operating at a scattering angle of 175°. The samples were prepared by mixing an appropriate amount of water, salt, surfactant, and 0.2 mL of 50 mg/L SL particle dispersion. The final volume (1 mL) and the SL particle concentration (10 mg/L) were kept constant in the experiments. The samples were left to rest for 2 h at room temperature. The electrophoretic mobility of each sample was measured five times, averaged, and converted to zeta potential (ζ) using the Smoluchowski equation^36^
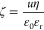
1where η is the viscosity of the medium, ε_0_ is the dielectric permittivity of vacuum, and ε_r_ is the relative permittivity of water. Furthermore, the surface charge density (σ) was calculated from the ionic strength dependence of the ζ data applying the Debye–Hückel model^37^
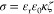
2where κ is the inverse Debye length, which quantifies the contribution of the background electrolyte concentration to the extension of the electrical double layer.^36^

### Dynamic Light Scattering

Particle aggregation was investigated with time-resolved dynamic light scattering (DLS) measurements using a NIBS High-Performance Particle Sizer (ALV) instrument equipped with a 633 nm He/Ne laser as a light source, while the scattering angle was 173°. The correlation functions were collected for 20 s, and 100 runs were performed for each time-resolved experiment. A second-order cumulant fit was applied to the correlation function to determine the hydrodynamic radius (*R*_h_).^38^ For the early stages of aggregation, the absolute aggregation rate constant (*k*) was calculated from the initial change of the *R*_h_ as a function of time (*t*) as follows^39,40^
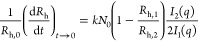
3where *R*_h,0_ is the initial hydrodynamic radius, *N*_0_ is the initial number concentration of the particles, and *R*_h,1_ and *R*_h,2_ correspond to the hydrodynamic radius of the monomer and dimer, respectively. The contribution of the form factors of the monomer (*I*_1_) and dimer (*I*_2_) to the scattered intensity was calculated with the theory developed by Rayleigh, Debye, and Ganswhile.^38^ The left side of eq 3 can be experimentally determined from the slopes of the apparent hydrodynamic radius versus time plots, as shown in Figure S7. The aggregation was further reported in terms of the stability ratio, which is the fast aggregation rate coefficient (*k*_fast_) divided by the one measured in the actual experiment (*k*)^38,39^
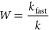
4

Note that to probe the early stages of aggregation, that is, where mainly particle monomers and dimers are present, the *R*_h,0_ values should agree within experimental error with *R*_h,1_, and the relative increase of *R*_h_ should be less than 40% of its initial value.^38^ Such conditions are best to be determined by varying the particle concentration (Figure S7a), and it was found that the optimal concentration is 10 mg/L for SL particles. This figure also indicates that the obtained slopes are proportional to the particle concentration, as can be predicted by eq 3. The DLS measurements were performed in disposable cuvettes (VWR), and the sample preparation was the same as in the electrophoretic study, with the exception that the aggregation experiments were started immediately after adding the particle dispersion to the samples and subsequently mixing.

## Results and Discussion

Charging and aggregation features of negatively charged SL particles were investigated by electrophoretic and time-resolved DLS measurements in the presence of GSs with different structures (Figure 1). The GS composition was systematically varied so that the influence of both the alkyl chain and spacer length could be addressed. In addition, the interfacial behavior of the 12-4-12 GS was assessed in more detail and compared to the conventional DTAB monomeric surfactant of the same hydrocarbon chain length. Finally, the effect of concentration and composition of inorganic electrolytes on the colloidal stability of the SL–GSs dispersions was systematically explored.

### Stability of SL Particles in the Presence of GSs

**Figure 2:**
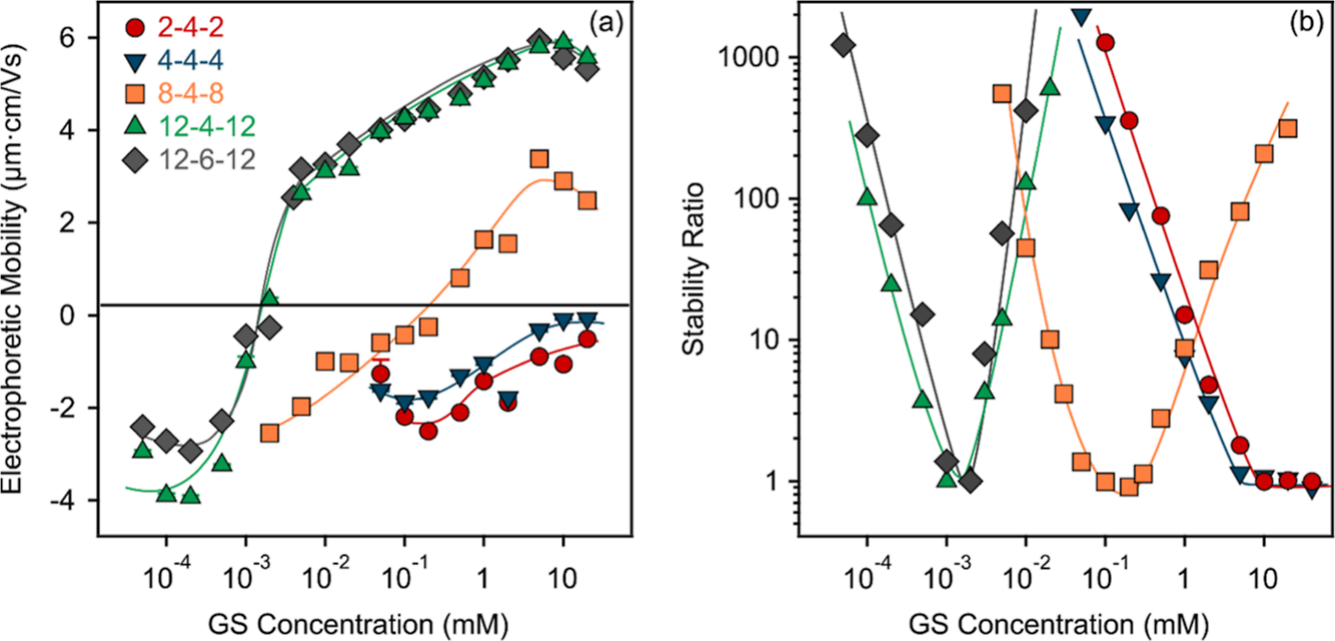
Electrophoretic mobility (a) and the stability ratio (b) of SL particles as a function of GS concentration. The solid lines serve to guide the eye only.

The main objective of this part of the investigations was to clarify the structural effects of GSs on the colloidal stability of the oppositely charged SL particle dispersions. The tendencies of electrophoretic mobilities of the latex particles in the presence of the varied amount of GS are shown in Figure 2a. At very low GS concentrations, the SL particles are negatively charged as expected based on their surface chemistry. The negative charge enables favorable interaction of the SL surface groups with the positively charged GS cations, and thus, the cations are electrostatically attracted to the charged surface sites. Consequently, after an intermediate minimum due to the electrokinetic effect,^41^ the mobility increases with the concentration for all GSs. In the case of 2-4-2 and 4-4-4, however, it remained negative in the whole concentration regime studied, as is typical for indifferent salt constituents.^42^ Although the rise in the mobilities and partial charge neutralization is mainly due to the surface charge screening by the cations, the slightly different mobilities indicate adsorption of 2-4-2 and 4-4-4 on the SL particles to a different extent, with the affinity of the latter one to the surface being somewhat higher.

However, for GSs of longer alkyl chains (8-4-8, 12-4-12, and 12-6-12), the adsorption behavior differs significantly from that of small molecules and ions, and the trend can be described by the reverse orientation model.^28,43^ Accordingly, surfactants adsorb already at low concentrations because of the electrostatic interactions between their charged head groups and the oppositely charged solid surface. Then, in parallel to the increasing surfactant concentration, the hydrophobic interactions between the hydrophobic tails of the adjacent adsorbed surfactant molecules also become significant. The combined impact of electrostatic and hydrophobic interactions, and the subsequent increase in the amount of adsorbed GSs, causes a tremendous rise in the mobilities until the SL particles are neutralized at a system-specific concentration, which is referred to as the isoelectric point (IEP). The determined IEP data are shown in Table 1. Above this concentration, the subsequent surfactant adsorption is driven by hydrophobic interactions; thus, the formation of surfactant bilayer structures at the surface of SL, with the heads of the second surfactant layer pointed toward the solution, results in the greatest positive mobility values. The ability to reverse the sign of surface charge is typical for polyelectrolytes or surfactants adsorbing on oppositely charged particles.^15,44^ Finally, the mobilities decrease at high GS concentrations owing to the charge screening effect of the bromide counterions of the surfactants.

One can also notice in Figure 2a that there is a shift in the IEP values, that is, it is about two orders of magnitude lower for the 12-4-12 and 12-6-12 compared to 8-4-8. Further comparison of the mobility curves reveals that the extent of the adsorption increased with increasing the length of the hydrophobic tails; that is, adsorption of the more hydrophobic GSs caused higher electrophoretic mobilities upon charge reversal. In contrast, the change in the spacer’s length had no significant effect on the charging characteristics.

The stability ratios investigated under the same experimental conditions (e.g., particle concentration, pH, and GS concentration range) as in the electrophoretic study mentioned above are shown in Figure 2b. *W* ∼ 1 corresponds to fast aggregation and an unstable sample, while *W* > 1 indicates slower aggregation, where the dispersion is more stable; that is, only a fraction of the particle collisions gives rise to the dimer formation. The general trend in the stability ratios was very similar in the cases of 2-4-2 and 4-4-4, which followed the prediction of the DLVO (Derjaguin, Landau, Verwey, and Overbeek) theory, indicating that the acting major interparticle forces are of electrostatic origin.^42,45^ Accordingly, at low GS concentration, the stability ratio is high, which is referred to as the slow aggregation regime. However, the value of the stability ratio decreases rapidly with increasing concentration until its value becomes one at higher doses, and this regime is referred to as the fast aggregation regime. These regions are separated by the critical coagulation concentration,^46^ which can be determined from the stability ratio versus GS concentration plots as follows^47^
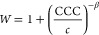
5where *c* is the molar concentration of the salt, and β was calculated from the slope of the stability ratio versus GS concentration curves before the CCC.

However, 8-4-8, 12-4-12, and 12-6-12 GSs show more complicated behavior, featuring the characteristic U-shaped stability plot, which has been reported for several particle systems containing oppositely charged polyelectrolytes or surfactants.^15,16,40,48,49^ The SL dispersions were stable at low surfactant concentrations, as indicated by the fairly high stability ratio values determined in this regime. Then, with increasing surfactant amount, the stability ratios decreased and reached a minimum, followed by an increase at higher concentrations, where stable samples were observed.

**Figure 3:**
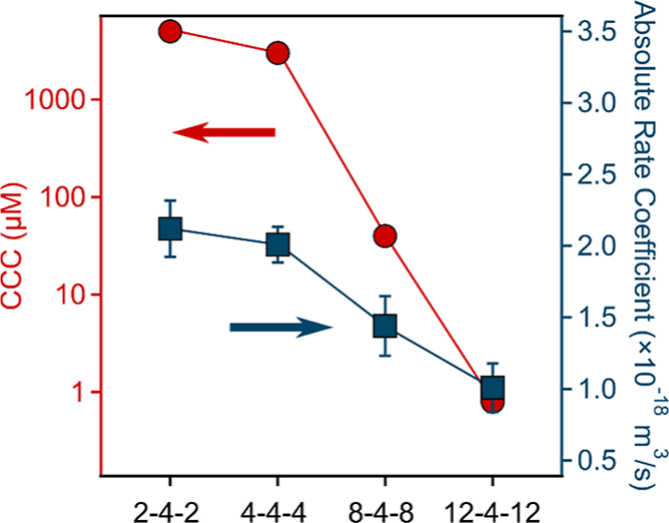
CCCs (left axis, calculated with eq 5) and absolute fast aggregation rate coefficients (right axis, calculated with eq 3) for SL particles in the presence of different GSs. The data were determined by averaging the aggregation rates obtained above the CCC in each system. The solid lines are eye guides.

A correlation between the charging behavior and the aggregation processes can be established by comparing the abovementioned tendencies in the stability curves with the electrophoretic mobility data. Accordingly, the minima of the stability ratios are located near the IEPs, whose tendencies can be rationalized within the DLVO theory. Indeed, once the surface charges are neutralized at the IEP, the SL particles (with adsorbed GSs on the surface) rapidly aggregate because the energy barrier vanishes due to the lack of electrical double layer forces leading to the predominance of the attractive van der Waals forces. However, when the particles possess a sufficiently high charge (either positive or negative), the electrostatic repulsion is strong enough to keep the particles apart. Another important aspect is that with increasing alkyl chain length, the destabilization power of the GSs also increases, that is, the CCC values decrease in the 2-4-2 > 4-4-4 > 4-8-4 > 12-4-12 order (Figure 3 and Table 1), in agreement with the tendency observed for the IEP data due to the above-discussed charge-aggregation relationship.

Similar charge-aggregation relations were reported earlier for systems containing colloidal particles and monomeric surfactants of different alkyl chain lengths. Accordingly, the affinity of alkyl sulfate surfactants to the surface of latex particles increased with the increasing length of the alkyl chain, which affected the onset of the fast aggregation regimes.^15^ The same phenomenon was observed for hematite particles.^17^ However, for ionic liquid cations, such a tendency was observed only for hydrophobic lattices,^50^ while the reversed trend, that is, the IEP and CCC values increased by increasing the alkyl chain length, was obtained for hydrophilic titania particles.^51^

In contrast, the length of the spacer had no effect on the colloidal stability. Since both electrophoretic mobilities (Figure 2a) and stability ratios (Figure 2b) were the same within the experimental error for both 12-4-12 and 12-6-12, one can conclude that the interfacial features of the GSs are independent of the distance between the head groups. This information also sheds light on the fact that the adsorption processes are mainly influenced by the aliphatic chain length and that the spacer are not involved in the GS assembly on the surface.

In addition, we also investigated in more detail the effect of the composition of GSs on the aggregation rates (calculated by eq 3) in the fast aggregation regimes. The data presented in Figure 3 and Table 1 show that the fast aggregation rate coefficients were also sensitive to the structure of the investigated GSs. It was found that with increasing alkyl chain length, the fast aggregation rate coefficient decreases, indicating the presence of additional non-DLVO forces. These findings suggest that the interparticle forces (e.g., van der Waals and possible hydrophobic interactions) are sensitive to the type of the GS. Since electrostatic interactions are fully screened at the IEP, the rapid aggregation is influenced by additional repulsive forces, which could originate from steric repulsion between the chains of the adsorbed surfactant layers.^52^ During the development of such repulsive forces, the overlap between the adsorbed GS and the resulting increase in osmotic pressure, which occurs when particles covered with the GS approach each other, play the most important role. This phenomenon has been widely investigated in the case of adsorbed polymer layers; however, in the presence of surfactants or GSs, only a few systematic studies can be found in the literature.^16^ Another possible explanation for such a tendency in the fast aggregation rate coefficients is related to the structure-dependent difference in the GS adsorption reversibility. Accordingly, GSs of short alkyl chains possess much less affinity to the particle surface, as reflected in the mobility data (Figure 2a). This leads to their quicker desorption, and thus, the steric stabilization force is weaker. In contrast, the possible desorption and rearrangement processes for the GSs of longer chains are much slower, which leads to their more pronounced stabilizing effect.

### Comparison of 12-4-12 to Its Monomeric Counterpart

To explore the differences and similarities in the interfacial behavior of dimeric and monomeric surfactants, the effect of 12-4-12 and DTAB on the colloidal stability of SL was compared. Although both surfactants have the same head group(s) and alkyl chain lengths, connecting two DTAB molecules at the level of head groups with a spacer may result in markedly different properties. The 12-4-12 GS is reported to be more surface active than DTAB, which is also supported by their different critical micelle concentration (CMC) data of 1.09 mM for 12-4-12 and 15.3 mM for DTAB.^28^

**Figure 4:**
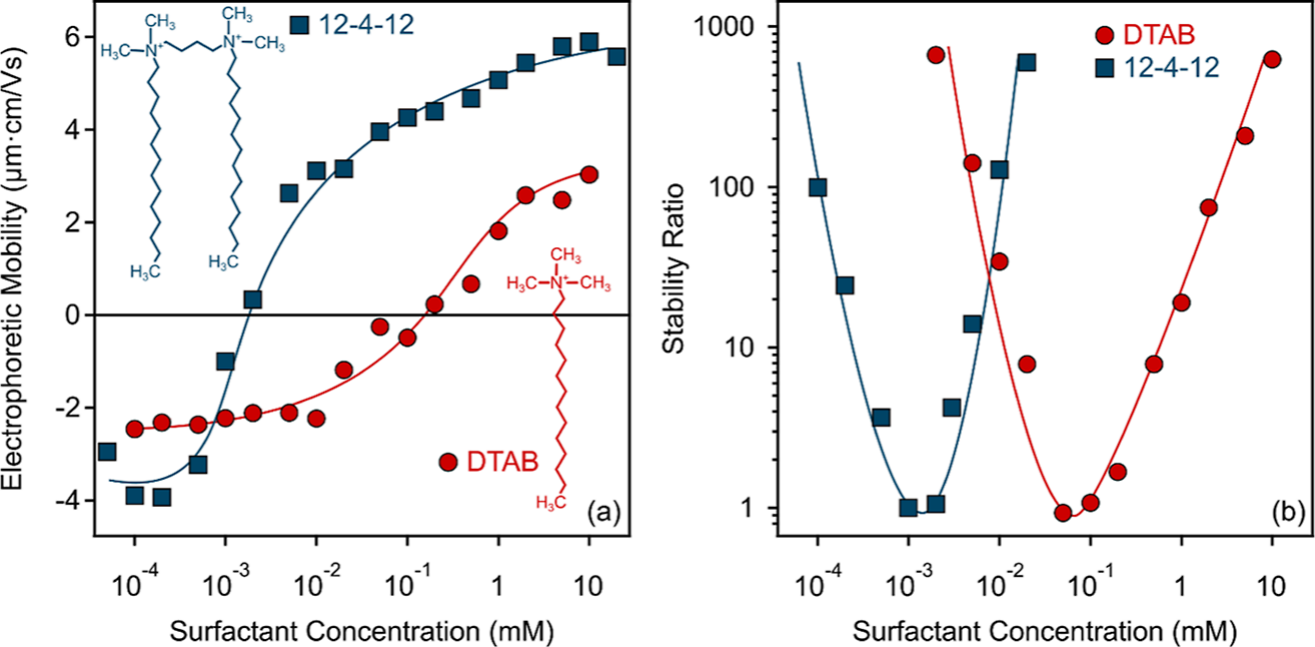
Comparison of the electrophoretic mobility (a) and stability ratio (b) data of SL particles in the presence of gemini (12-4-12) and conventional monomeric (DTAB) surfactants of the same aliphatic chain length. The solid lines serve to guide the eye only. The structures of 12-4-12 and DTAB are presented in the inset.

For comparison, the electrophoretic mobility and stability ratio values were determined in the presence of DTAB under the same experimental conditions as in the case of GSs. Figure 4 indicates that the overall charging and aggregation tendencies are similar in the presence of conventional monomeric and GSs, except there is a shift by about two orders of magnitude to lower concentrations in the IEP for the 12-4-12 compared to DTAB, which is in line with the extracted CCC values (Table 1). The formation of surfactant bilayers at concentrations lower than the CMC demonstrates the high adsorption affinity of the selected surfactants to the SL surface, as shown previously in other dispersed systems too.^16,34^ Besides, the fact that the IEP of the 12-4-12 is not half of that of DTAB’s IEP (as one would assume from the concentration of the individual head groups) can be attributed to the stronger hydrophobic interaction between the tails in systems containing the dimeric surfactant of two dodecyl chains. Therefore, it can be concluded that lower concentrations of 12-4-12 can be used either for the stabilization or destabilization of SL particles compared to the monomeric counterpart.

Besides, two other observations can be made based on the data presented in Figure 4, which deserves further discussion. First, the highest electrophoretic mobilities measured above the IEP were +5.8 and +3.0 μm·cm/V s for 12-4-12 and DTAB, respectively. This result indicates that 12-4-12 not only reaches the IEP at lower concentrations, but its adsorption leads to the development of considerably higher positive surface charges, which increases the electrostatic interparticle repulsion between the particles, that is, a more stable colloidal system may be formed. Second, the fast aggregation coefficient value determined for 12-4-12 is higher than for DTAB (Table 1). This finding points out that near the IEP, where no electrostatic repulsion exists between the particles, stronger steric repulsion takes place between the chains of the adsorbed DTAB upon the approach of two particles than in the presence of 12-4-12. These are highly valuable experimental information, while the unambiguous explanation of the underlying phenomena and mechanisms requires further studies.

### Effect of Salinity on the Stability of SL-GSs Dispersions

The applicability of particle dispersions in the presence of surfactants is strongly influenced by factors such as ionic strength and background electrolyte composition.^53^ Thus, the colloidal stability of the SL-12-4-12 system was explored in wide concentration ranges of various inorganic salts. In this way, the basic colloidal properties of the bare SL particles in terms of the influence of salt composition and valence of the counter and coions were assessed first. Accordingly, KCl, K_2_SO_4_, and CaCl_2_ salts were used, and the obtained electrophoretic mobility and stability ratio data were analyzed.

**Figure 5:**
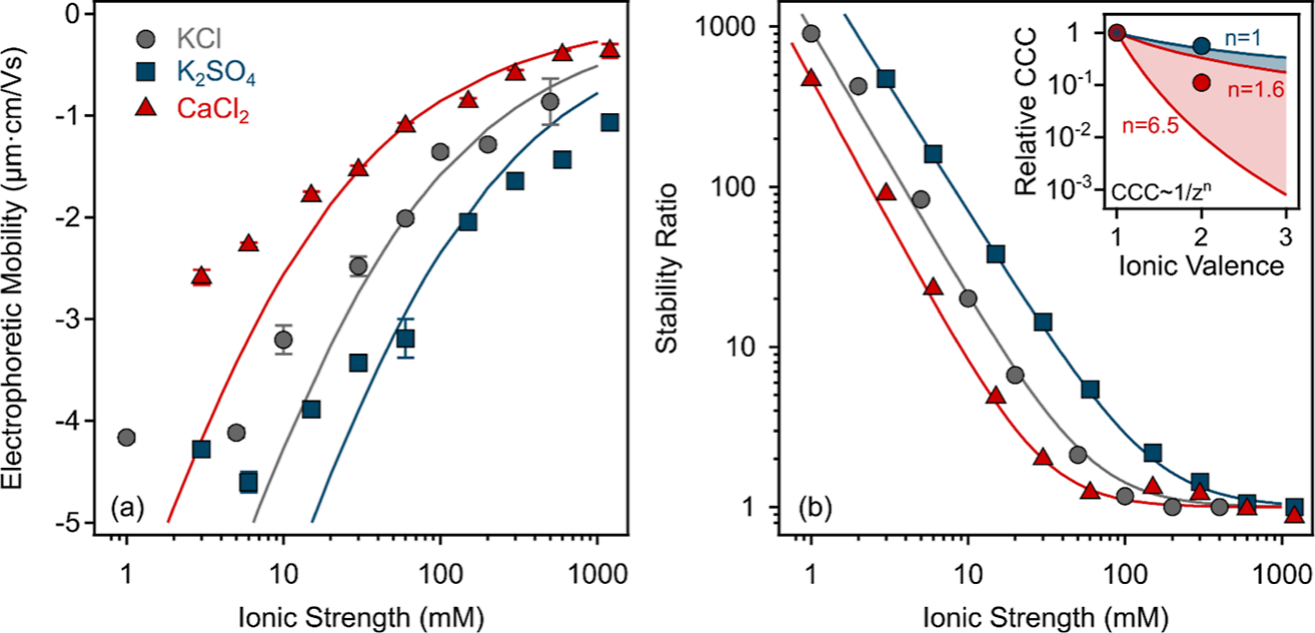
Electrophoretic mobilities (a) and stability ratios (b) of SL particles as a function of the ionic strength using KCl, K_2_SO_4_, and CaCl_2_ salts. The solid lines in (a,b) were obtained using eqs 2 and 5, respectively. The inset in (b) shows the relative CCC values (normalized to the CCC obtained for KCl) as a function of the ionic valence. The solid lines in the inset indicate the direct (*n* = 1.6 and 6.5 in eq 7) and the inverse (*n* = 1 in eq 7) Schulze–Hardy rule.

The ionic strength-dependent mobility values are shown in Figure 5a. In general, the shape of the obtained plots is similar regardless of the salt composition, that is, the absolute values of the mobilities decreased with the increasing ionic strength due to charge screening by the salts, in line with the classical models used to describe the salt-dependent behavior of the electrical double layer.^36^ However, owing to specific ion adsorption, the mobility values at a given ionic strength significantly differ and it follows the CaCl_2_ >KCl > K_2_SO_4_ order. The surface charge densities (Table S2), determined from the ionic strength dependent potential data using eq 2, decreased in the same order, indicating system specific surface–ion interactions.

Tendencies in the data in Figure 5b indicate a typical dependence of the stability ratios on the ionic strength in the presence of all inorganic salts. Some characteristic time-resolved DLS curves for KCl are shown in Figure S7b. The obtained critical coagulation ionic strength (CCIS) values decreased in the K_2_SO_4_ > KCl > CaCl_2_ order (Table S2), in accordance with the surface charge densities. Comparing the charge density and CCIS values in the presence of KCl and CaCl_2_, the higher affinity of the Ca^2+^ to the negatively charged particle is clearly visible since these parameters decreased significantly as the counterion’s valence was increased. This finding can be explained by the fact that divalent cations are more effective in charge screening than monovalent ones, as reported earlier in other particle–electrolyte systems.^11,18,53−56^

For further data evaluation, CCISs were converted to CCCs (Table S2) using the ionic strength (*I*) as
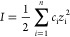
6where *c*_*i*_ is the molar concentration of ion *i* (mol/L), *z*_*i*_ is the charge number of that ion, and the sum is taken over all ions present in the solution. The obtained tendency in the destabilization power is compared to the Schulze–Hardy rule, which states that the CCC dependence on the ionic valence (*z*) can be quantified as^37,55^
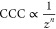
7where the value of *n* is determined by the surface charge and it is also related to the hydrophobicity of the particles. When considering asymmetric electrolytes, the exponent for particles with a high surface charge is *n* = 6.5; however, for weakly charged particles. it tends to be *n* = 1.6. The inset in Figure 5b shows the relative CCCs normalized to the CCC obtained in the presence of KCl, as well as the CCC values expected from the Schulze–Hardy rule (eq 7) with the abovementioned limits. The obtained data for CaCl_2_ appear between these limits, which indicate that SL particles have an intermediate surface charge.

However, once multivalent ions (SO_4_^2–^) represent the coions (e.g., same sign of charge as the particle surface), the CCIS increases by increasing the valence of the anion. Furthermore, when CCIS was converted to CCC, a reversed tendency could be observed, that is, the CCC slightly decreases with the increasing ionic valence of the coion. This tendency is in accordance with the inverse Schulze–Hardy rule,^54,57^ which predicts that the dependence of the CCC on the coion valence is much less significant (*n* = 1 in eq 7), as in the case of multivalent counterions. The obtained result for SO_4_^2–^ is in good quantitative agreement with the prediction of the rule.

**Figure 6:**
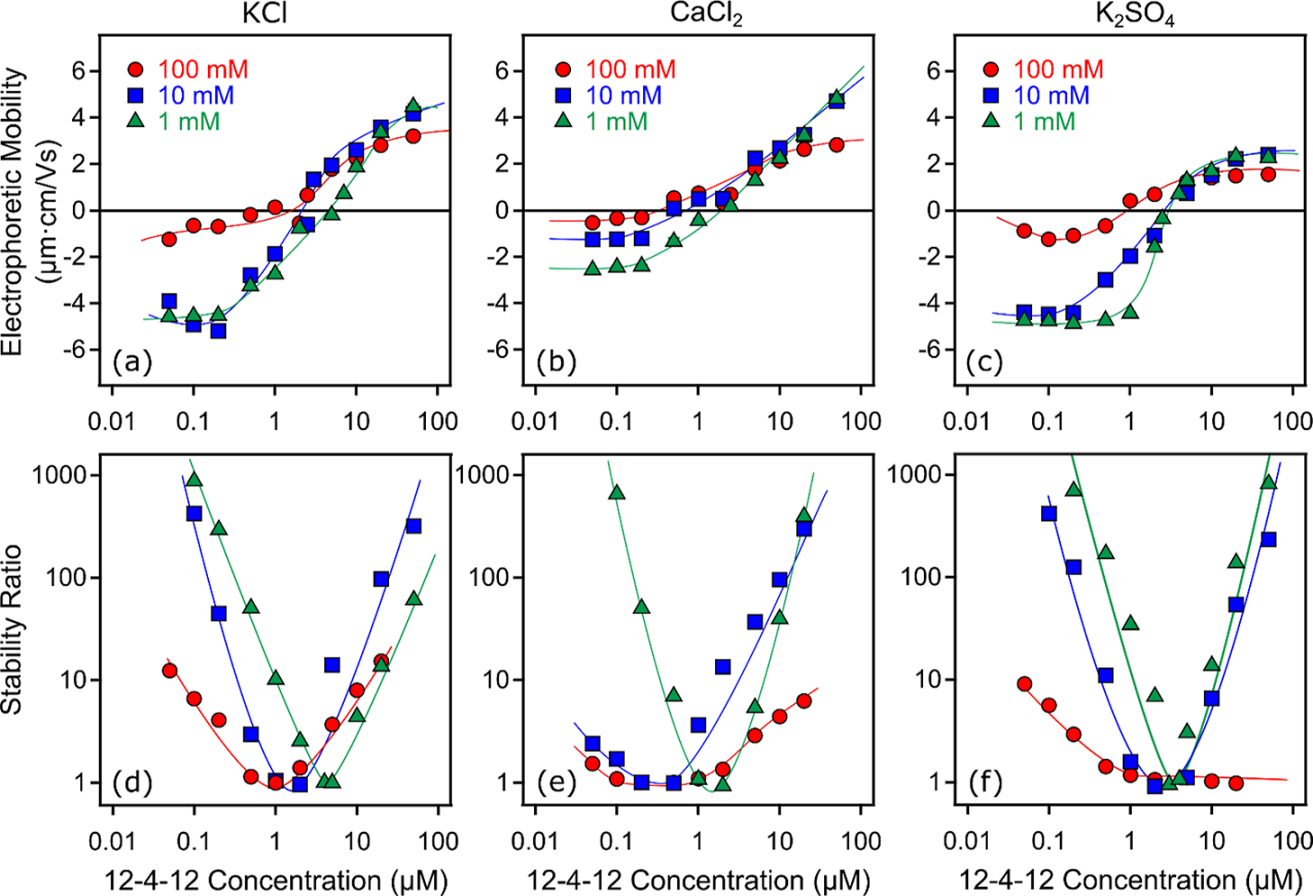
Electrophoretic mobility (a–c) and stability ratio (d–f) of SL particles as a function of 12-4-12 concentration in the presence of KCl (a,d), CaCl_2_ (b,e), and K_2_SO_4_ (c,f) at different concentrations (1, 10, and 100 mM). The solid lines serve to guide the eyes.

After the basic colloidal characterization of the SL particles in the presence of inorganic salts, the 12-4-12 surfactant was also introduced in the systems (Figure 6). The charging properties of SL particles in the presence of 12-4-12 at different salt concentrations (1, 10, and 100 mM) are shown in Figure 6a–c.

When the concentration of the background salt solution is increased, the qualitative behavior remains the same as in the systems without the added salt (Figure 2a), albeit the magnitude of the mobilities is reduced due to additional screening, and the exact values were also sensitive to the composition of the electrolyte applied. Furthermore, with the increasing ionic strength, the IEP shifts toward lower 12-4-12 concentrations, showing that the surfactant and the salt constituent ions compete for adsorption sites.

The second noteworthy aspect regarding the charging features is that the ionic valence also had a remarkable impact on the overall results. Increasing the valence of the ions, which have the opposite sign of charge as the particles under the given experimental conditions, always showed more pronounced effects. Therefore, at low 12-4-12 concentrations, where the particle possesses a negative surface charge, Ca^2+^ ions had the most remarkable effect, that is, the particle’s charge was reduced the most in this case. In contrast, at high 12-4-12 concentrations, where the charge of the particles was positive due to charge reversal, the SO_4_^2–^ ions decreased the mobilities to the highest extent since it acts as a counterion under these experimental conditions.

In addition, the effect of the salt composition on the colloidal stability was also investigated in the SL-12-4-12 by time-resolved DLS (Figure 6d–f). In general, these data again exhibit U-shaped stability plots in most cases, corresponding to the charge reversal process. However, the pronounced salt dependence of the stability ratio values can be observed in the individual systems. The data indicate that near the IEP, the aggregation is rapid, as predicted based on the DLVO theory. When moving away from the IEP in either direction parallel to the increasing surface charge, higher stability ratios can indicate stronger repulsion. However, the ionic strength has a profound effect on the stability curve, and hence, the typical trend, irrespective of the salt composition, is that at low ionic strength, the fast aggregation regime is very narrow, but widens significantly with the increasing ionic strength. This tendency can be explained by the decreased electrostatic repulsion between the particles, owing to the enhanced charge screening at higher salt levels. This effect can be qualitatively rationalized within the DLVO theory, and it was also observed for charged colloidal particles in the presence of polyelectrolytes.^44^

Comparing the trend in the mobilities and stability ratios, one can easily realize that, when the particles possess net negative charge, that is, at low 12-4-12 concentrations, the destabilization power (quantified with the CCC) of the salts follows the same KCl > K_2_SO_4_ > CaCl_2_ order (Figure S8 and Table S3), as in the case of the bare SL (Figure 5b). These observations indicate that the tendency in this regime is dictated by the characteristics of the bare particle surface. Note that the CCC values in the presence of 10 and 100 mM CaCl_2_ could not be determined since the SL particles already tend to aggregate at these CaCl_2_ concentrations. Furthermore, the IEP values are systematically higher than the CCC values, implying that the onset of the decrease in mobility correspond to the onset of destabilization, rather than to reaching complete charge neutralization at the IEP.^58^

**Figure 7:**
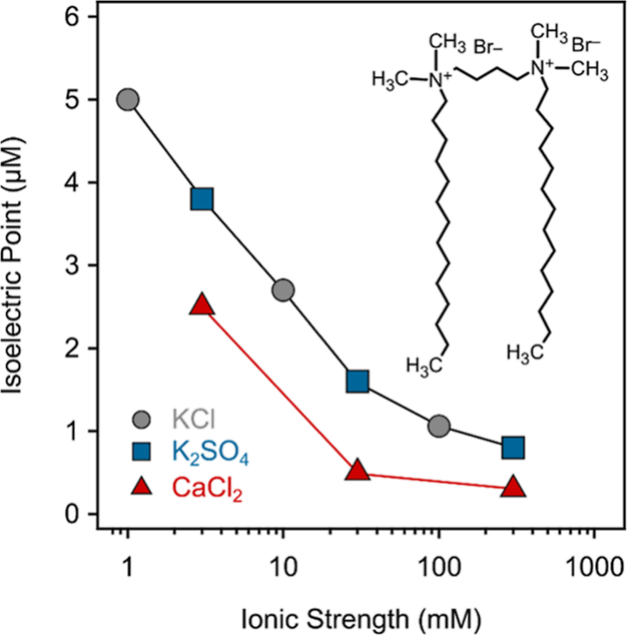
IEPs as a function of the ionic strength in the presence of KCl, CaCl_2_, and K_2_SO_4_ measured for SL-12-4-12 dispersions. The inset shows the structure of the 12-4-12 surfactant.

Another important feature is that the IEP values in the case of K_2_SO_4_ fit in the tendency of IEP in the presence of KCl, if we consider the ionic strength (Figure 7 and Table S3). This fact indicates that in comparison with Cl^–^, SO_4_^2–^ ions had no specific effect at low GS concentrations, where these ions act as coions. In contrast, the Ca^2+^ ions had a significant impact on the IEP value, since they were the counterions below the charge neutralization, and thus, their influence is more pronounced in this regime. Under these experimental conditions, competition can be assumed between the surfactant and Ca^2+^ ions for surface adsorption sites; therefore, fewer 12-4-12 molecules are required to neutralize the surface charge of SL. However, above the IEP, where the particle’s net charge becomes positive, an inverse trend can be observed. In this situation, the SO_4_^2–^ ions behave as counterions, and their surface adsorption on the surfactant-covered particles can be assumed. Due to their charge neutralizing effect expressed in this way, dispersions were also destabilized at sufficiently high ionic strengths (Figure 6f). In contrast, KCl and CaCl_2_ had very similar, but significantly lower effects in this 12-4-12 concentration regime.

Based on the abovementioned results, one can conclude that the colloidal stability of the SL-12-4-12 dispersions is heavily influenced by the chemical composition and concentration of the dissolved salts present. Since basic research and industrial processes, in which GSs and particles are applied, often involve background electrolytes, these results provide valuable information for the design of processable colloid systems.

## Conclusions

The colloidal stability of SL particles of the negative surface charge was studied by light scattering techniques in the presence of oppositely charged GSs. The ones of short alkyl chains (2-4-2 and 4-4-4) destabilize the colloidal dispersion by charge screening, similar to simple inorganic ions. However, GSs of longer alkyl chains (8-4-8, 12-4-12, and 12-6-12) lead to destabilization by charge neutralization, which is followed by subsequent restabilization due to overcharging. It was found that the length of the aliphatic tails of GSs plays a significant role in the alteration of the colloidal stability of SL particles, and that the CCCs followed the 2-4-2 > 4-4-4 > 8-4-8 > 12-4-12 order, which correlates to the charging properties. Comparing the interfacial features of 12-4-12 GS with the corresponding monomeric surfactant (DTAB) reveals that the dimeric surfactant has a much higher adsorption affinity to the particle surface, and thus, both destabilization and stabilization of the SL particles require lower doses of added 12-4-12 compared to DTAB. The adsorption processes in the SL-GSs systems are controlled by electrostatic attraction through the head groups as well as by hydrophobic interactions via the aliphatic chains. Interparticle forces involved in determining the colloidal stability were described within the DLVO theory, while the presence of additional nonelectrostatic forces, which originate from steric repulsion between the alkyl chains of surfactants adsorbed on the surface of the SL particle, was also observed. The background salt composition and the ionic strength strongly influenced the colloidal stability of SL through screening the electrostatic interactions. Divalent counterions showed a more pronounced destabilization effect for both SL and SL-GSs systems compared to divalent coions. Furthermore, with the increase in the ionic strength, the width of the fast aggregation regime became larger, while the CCC and IEP values shifted toward lower GS concentrations, indicating that the GS and the salt constituent counterions compete for the adsorption sites. The results help predict the behavior of GSs at the solid–liquid interfaces under different experimental conditions, which affects the colloidal stability, and thus, it can be especially useful to screen for surfactants in certain applications.

## Supporting Information Available

Experimental protocols, NMR spectra, thermograms, hydrodynamic radius, CCC, CCIS, IEP, and surface charge density data (PDF)
