# Supplementary material for: Structure–Stability Relationship in Aqueous Colloids of Latex Particles and Gemini Surfactants
Source: J Phys Chem B. 2022 Oct 26;126(44):9095–104. doi: 10.1021/acs.jpcb.2c06259 (PMC9910321; doi:10.1021/acs.jpcb.2c06259)
Supplement: Supplementary file 1 — jp2c06259_si_001.pdf [file jp2c06259_si_001.pdf]

Supporting Information (SI)

Structure-Stability Relationship in Aqueous Colloids of Latex Particles and Gemini Surfactants

Dóra Takács,[†] Tamás Péter,[†] Zsófia Vargáné Árok,[†] Bojana Katana,[†] Snežana Papović,[‡] Slobodan Gadzuric,[‡] Milan Vraneš,[‡] and István Szilágyi[†]*

[†]MTA-SZTE Lendület Biocolloids Research Group, Department of Physical Chemistry and Materials Science, University of Szeged, 6720 Szeged, Hungary

[‡]Department of Chemistry, Biochemistry and Environmental Protection, Faculty of Sciences, University of Novi Sad, 21 000 Novi Sad, Serbia

*Corresponding Author. Email: szistvan@chem.u-szeged.hu

METHODS

Nuclear Magnetic Resonance (NMR) Spectroscopy. The NMR spectra were recorded in D_2O at 298.15 K on a Bruker Advance III 400 MHz spectrometer. Tetramethylsilane was used as an accepted internal standard for calibrating chemical shifts for ^{1}H and ^{13}C. ^{1}H homo-decoupling and 2D COSY methods were used routinely for the assignation of obtained NMR spectra. ^{13}C NMR was assigned by the selective decoupling technique. The structure confirmation was carried out by ^{1}H and ^{13}C NMR analysis.

Thermogravimetric Measurements. The thermal stability of newly synthesized gemini surfactants was determined by thermogravimetric analysis (TGA) using simultaneous TG/DSC thermal analyzer SDT Q600 (TA Instruments, USA), with calorimetric accuracy and precision of ±0.2 % (based on metal standards) and temperature accuracy of ±0.5 K. The measurements were carried out under nitrogen atmosphere (a flow rate of 100 mL/min was applied) with samples placed in an open alumina pan (≈ 3.0 mg) up to 500 °C. The applied heating rate was 10 °C/min. The instrument was calibrated (for temperature and enthalpy) using an indium standard.

The obtained TG curves (Figure S6a) show that all synthesized salts are stable up to a temperature of at least 221.4 °C, indicating their remarkable thermal stabilities. The thermograms indicate weight loss by solvent evaporation near 100 °C for 2-4-2, demonstrating that this GS is significantly hygroscopic. The onset of thermal decomposition temperatures (T_{onset}) in Table 1 shows that the thermal stability decreases with the increase of the hydrocarbon chain length. No considerable effect of the length of the spacer on thermal stability was observed. However, the somewhat lower T_{onset} value for 12-4-12 in comparison to 12-6-12 suggests that increasing the length of the spacer slightly increases the thermal stability of GSs. Simultaneous DSC thermograms were recorded and presented in Figure S6b. It was found that only GS 4-4-4 possess a clear melting point at a temperature of 150 °C, while the other GSs were melting in parallel with thermal degradation.

a)

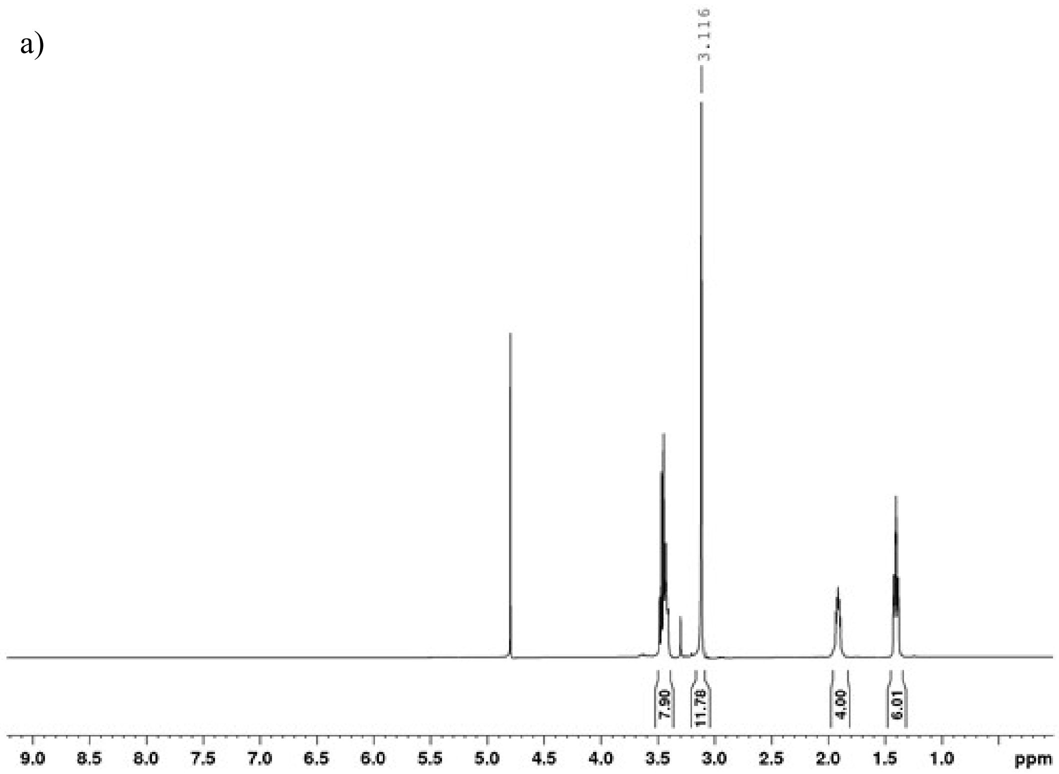

b)

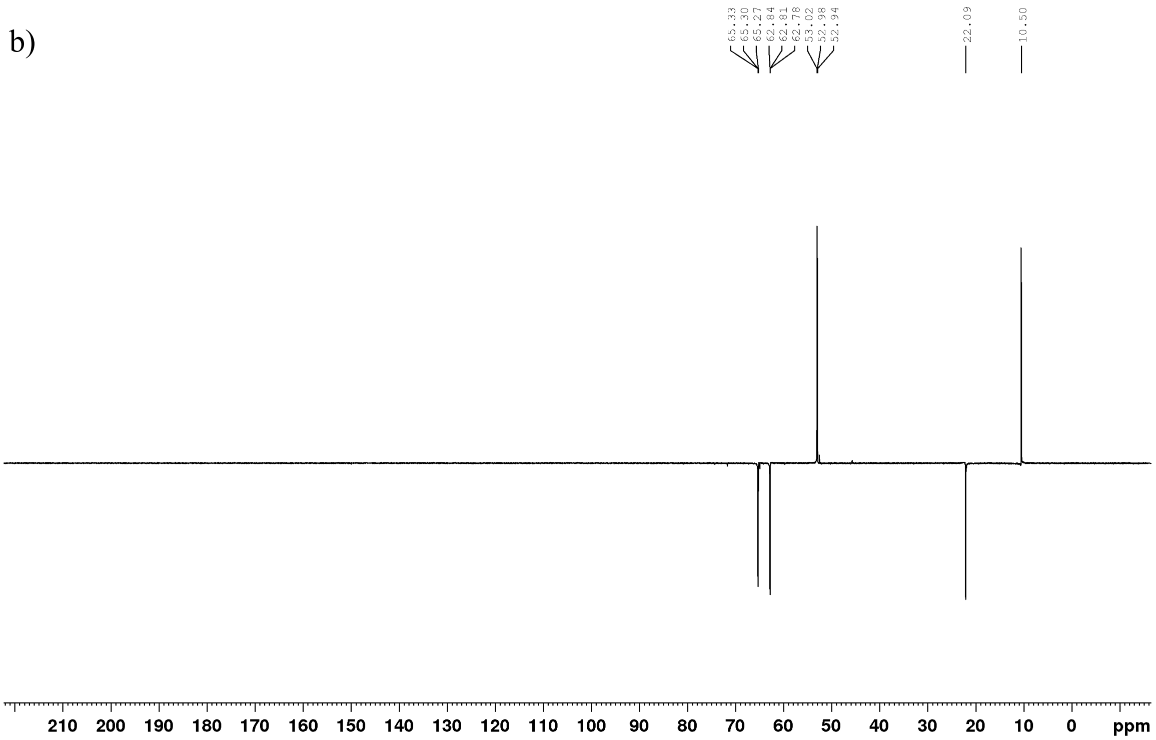

Figure S1. a) ^{1}H and b) ^{13}C NMR spectra of the synthesized *N*,*N′*-diethyl-*N*,*N*,*N′*,*N′*-tetramethylbutane-1,4-diammonium dibromide (2-4-2).

a) ^{1}H NMR (400 MHz, ppm, D_2O), 1.40, tt, 6H, $J_{CH2,CH3}$=7.3 Hz, J =1.8 Hz,2CH_3 from 2CH_2CH_3,

1.91, m, 4H, 2 NCH_2CH_2,

3.12, m, 12H, 2N$(CH_3)_2$,

3.39-3.50, m, 8H, 2 NCH_2CH_2, 2CH_2CH_3.

b) ^{13}C NMR (101 MHz, ppm, D_2O), 10.50 (CH_2CH_3),

22.09 (NCH_2CH_2),

52.94, 52.98, 53.02 (NCH_3),

62.78, 62.81, 62.84 (CH_2CH_3),

65.27, 65.30, 65.33 (NCH_2CH_2).

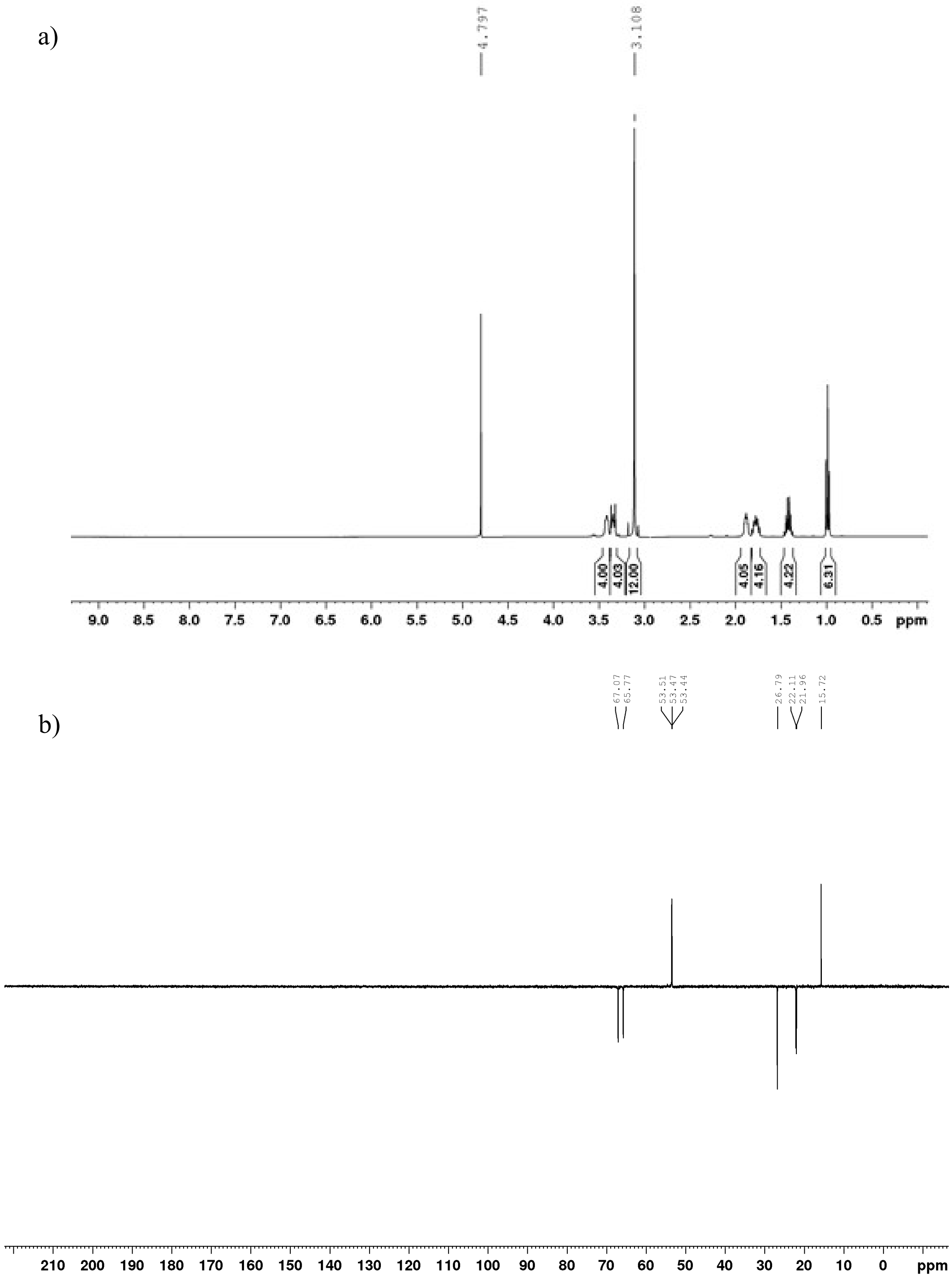

Figure S2. a) ^{1}H and b) ^{13}C NMR spectra of the synthesized *N*,*N′*-dibutyl-*N*,*N*,*N′*,*N′*-tetramethylbutane-1,4-diammonium dibromide (4-4-4).

a) ^{1}H NMR (400 MHz, ppm, D_2O), 0.99, t, 6H, $J_{CH2,CH3}$=7.3 Hz, 2CH_3 from 2N$CH_2CH_2CH_2CH_3$,

1.42, m, 4H, 2NCH$_2$CH$_2$CH_2CH$_3$,

1.77, m, 4H, 2NCH$_2$CH_2CH$_2$CH$_3$,

1.88, m, 4H, NCH$_2$CH_2CH_2CH$_2$N,

3.11, m, 12H, 2N(CH_3)$_2$,

3.34, m, 4H, 2NCH_2CH$_2$CH$_2$CH$_3$,

3.41, m, 4H, NCH_2CH$_2$CH$_2$CH_2N.

b) ^{13}C NMR (101 MHz, ppm, D_2O), 15.72 (NCH$_2$CH$_2$CH$_2$$CH_3$),

21.96 (NCH$_2$CH$_2$$CH_2CH_3$),

22.11 (NCH$_2$$CH_2$$CH_2CH_2$N),

26.79 (NCH$_2$$CH_2CH_2CH_3$),

53.44, 53.47, 53.51 (NCH$_3$),

65.77 (NCH$_2$CH$_2$CH$_2$$CH_2$N),

67.07 (NCH$_2$CH$_2$CH$_2$CH$_3$).

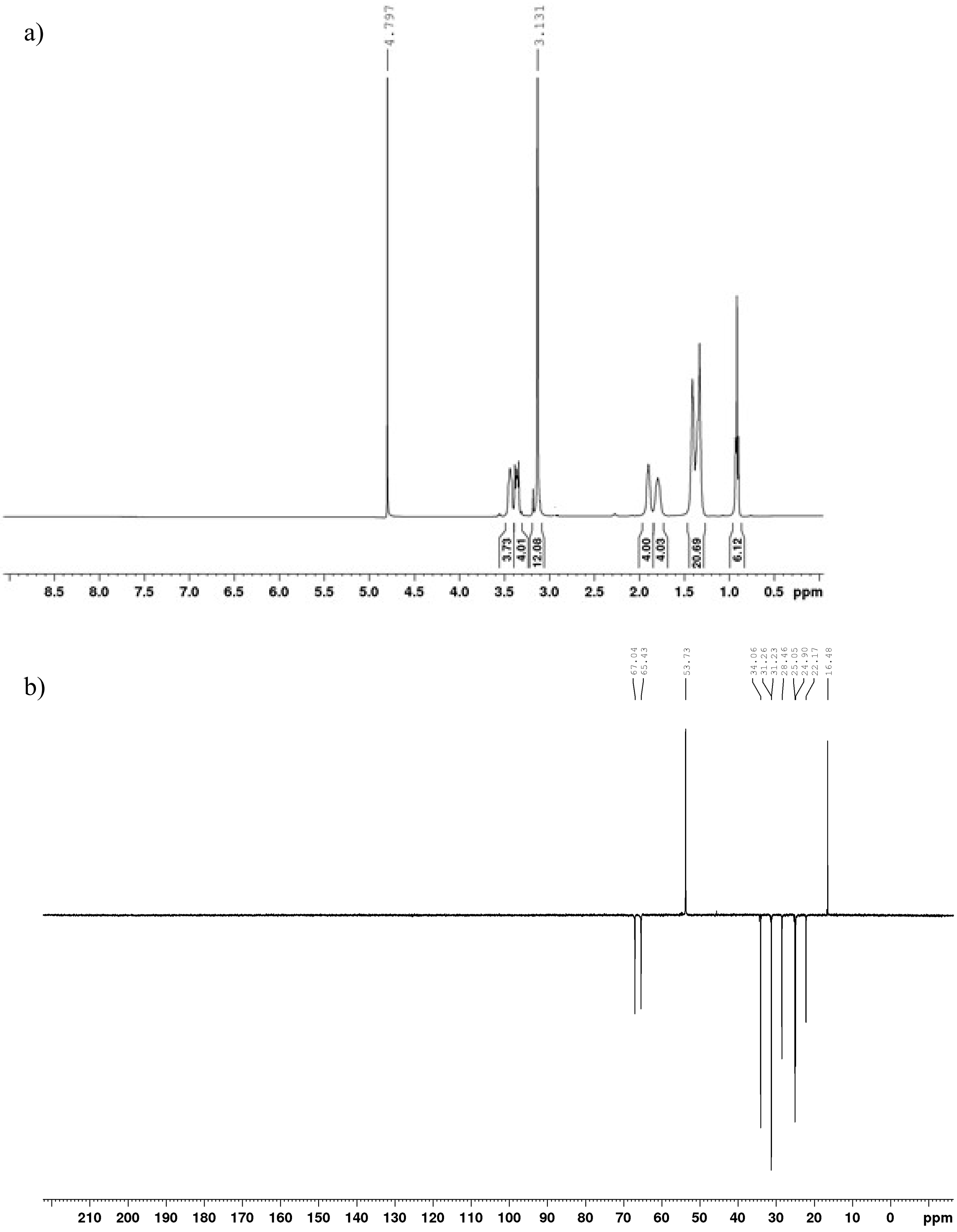

Figure S3. a) ^{1}H and b) ^{13}C NMR spectra of the synthesized *N*,*N*,*N′*,*N′*-tetramethyl-*N*,*N′*-dioctylbutane-1,4-diammonium dibromide (8-4-8).

a) ^{1}H NMR (400 MHz, ppm, D_2O), 0.91, t, 6H, $J_{CH2,CH3}$=7.0 Hz, 2CH_3 from $2NCH_2CH_2CH_2CH_2CH_2CH_2CH_2C\mathit{H}_3$,

1.28-1.42, m, 20H, $2NCH_2CH_2C\mathit{H}_2C\mathit{H}_2C\mathit{H}_2C\mathit{H}_2C\mathit{H}_2CH_3$,

1.77, m, 4H, $2NCH_2C\mathit{H}_2CH_2CH_2CH_2CH_2CH_2CH_3$,

1.79, m, 4H, $NCH_2C\mathit{H}_2C\mathit{H}_2CH_2N$,

3.13, s, 12H, $2N(C\mathit{H}_3)_2$,

3.36, m, 4H, $2NC\mathit{H}_2CH_2CH_2CH_2CH_2CH_2CH_2CH_3$,

3.44, m, 4H, $NC\mathit{H}_2CH_2CH_2C\mathit{H}_2N$.

b) ^{13}C NMR (101 MHz, ppm, D_2O), 16.48 ($NCH_2CH_2CH_2CH_2CH_2CH_2CH_2\mathit{C}H_3$),

22.17 ($NCH_2\mathit{C}H_2\mathit{C}H_2CH_2N$),

24.90 ($NCH_2\mathit{C}H_2CH_2CH_2CH_2CH_2CH_2CH_3$),

25.05, 28.46, 31.23, 31.26, 34.06 ($NCH_2\mathit{C}H_2CH_2CH_3$),

53.73 ($N\mathit{C}H_3$),

65.43 ($N\mathit{C}H_2CH_2CH_2\mathit{C}H_2N$),

67.04 ($N\mathit{C}H_2CH_2CH_2CH_3$).

a)

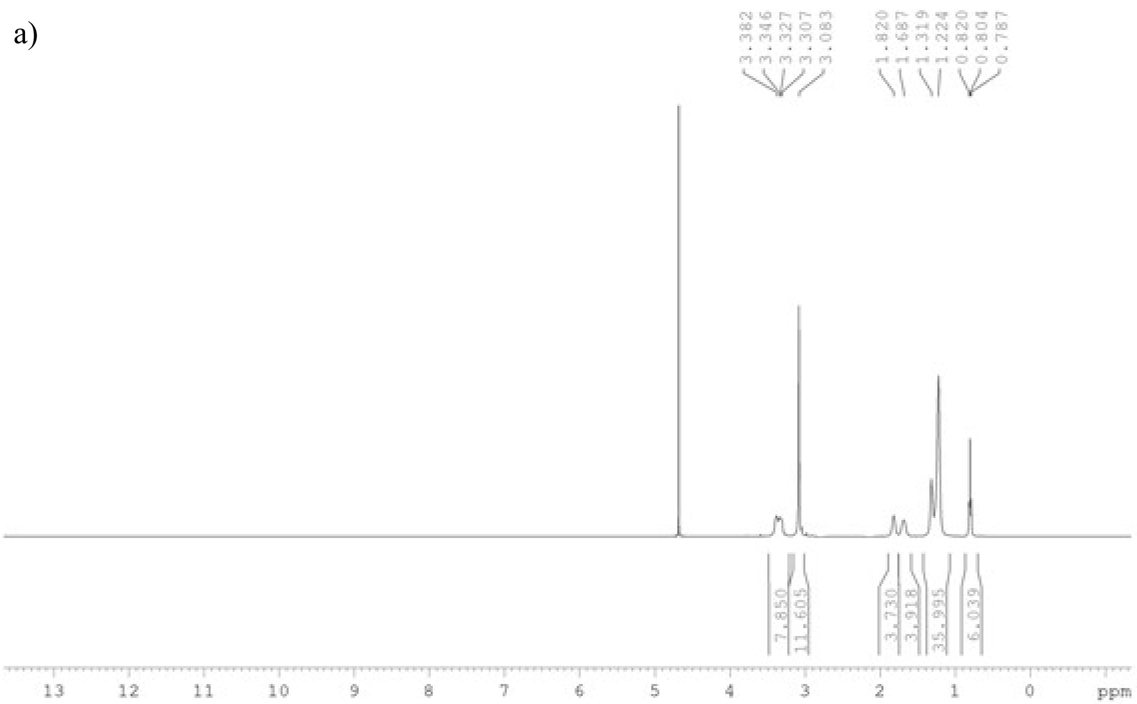

b)

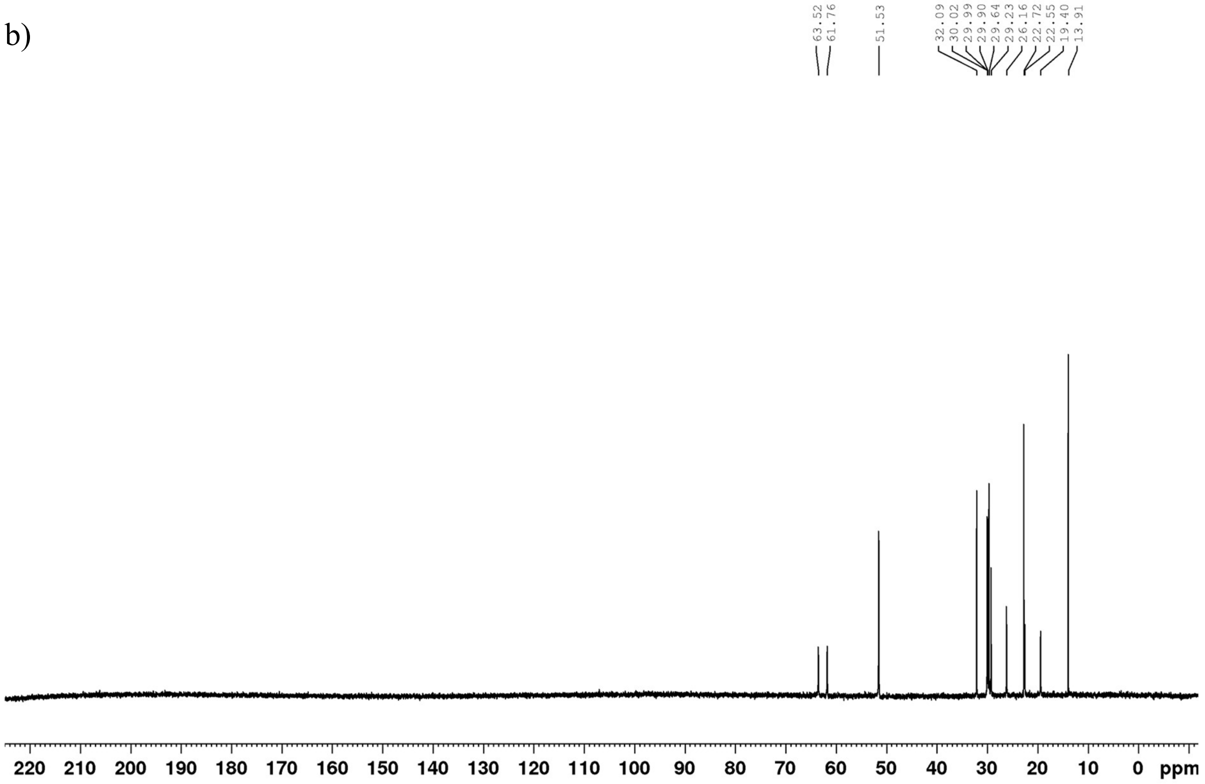

Figure S4. a) ^{1}H and b) ^{13}C NMR spectra for synthesized *N*,*N′*-didodecyl-*N*,*N*,*N′*,*N′*-tetramethylbutane-1,4-diammonium dibromide (12-4-12).

a) ^{1}H NMR (400 MHz, ppm, D_2O):

0.80, t, 6H, $\mathrm{2C}\mathit{H}_3$ from $\mathrm{2NCH_2CH_2CH_2CH_2CH_2CH_2CH_2CH_2CH_2CH_2CH_2C}\mathit{H}_3$,

1.69, m, 40H, $\mathrm{2NCH_2CH_2C}\mathit{H}_2\mathrm{C}\mathit{H}_2\mathrm{C}\mathit{H}_2\mathrm{C}\mathit{H}_2\mathrm{C}\mathit{H}_2\mathrm{C}\mathit{H}_2\mathrm{C}\mathit{H}_2\mathrm{C}\mathit{H}_2\mathrm{C}\mathit{H}_2\mathrm{CH_3}$,

$\mathrm{NCH_2CH_2C}\mathit{H}_2\mathrm{C}\mathit{H}_2\mathrm{CH_2CH_2N}$,

1.82, m, 4H, $\mathrm{2NCH_2C}\mathit{H}_2\mathrm{CH_2CH_2CH_2CH_2CH_2CH_2CH_2CH_2CH_2CH_3}$,

$\mathrm{NCH_2C}\mathit{H}_2\mathrm{CH_2CH_2C}\mathit{H}_2\mathrm{CH_2N}$,

3.08, s, 12H, $\mathrm{2N(C}\mathit{H}_3\mathrm{)_2}$,

3.24-3.45, m, 8H, $\mathrm{2NC}\mathit{H}_2\mathrm{CH_2CH_2CH_2CH_2CH_2CH_2CH_2CH_2CH_2CH_2CH_3}$,

$\mathrm{NC}\mathit{H}_2\mathrm{CH_2CH_2CH_2CH_2C}\mathit{H}_2\mathrm{N}$,

b) ^{13}C NMR (101 MHz, ppm, D_2O): 13.91

$\mathrm{(NCH_2CH_2CH_2CH_2CH_2CH_2CH_2CH_2CH_2CH_2CH_2}\mathit{C}\mathrm{H_3)}$,

19.40; 22.55; 22.72; 26.16; 29.23; 29.64; 29.90 $\mathrm{(N}\mathit{C}\mathrm{H_2}\mathit{C}\mathrm{H_2}\mathit{C}\mathrm{H_2CH_2N)}$,

$\mathrm{(NCH_2}\mathit{C}\mathrm{H_2}\mathit{C}\mathrm{H_2}\mathit{C}\mathrm{H_2}\mathit{C}\mathrm{H_2}\mathit{C}\mathrm{H_2}\mathit{C}\mathrm{H_2}\mathit{C}\mathrm{H_2}\mathit{C}\mathrm{H_2}\mathit{C}\mathrm{H_2}\mathit{C}\mathrm{H_2CH_3)}$

29.99; 30.02; 32.09; 51.53 $\mathrm{(N}\mathit{C}\mathrm{H_3)}$,

61.76, 65.75 $\mathrm{(N}\mathit{C}\mathrm{H_2CH_2CH_2CH_2CH_2}\mathit{C}\mathrm{H_2N)}$,

$\mathrm{(N}\mathit{C}\mathrm{H_2CH_2CH_2CH_2CH_2CH_2CH_2CH_2CH_2CH_2CH_2CH_3)}$.

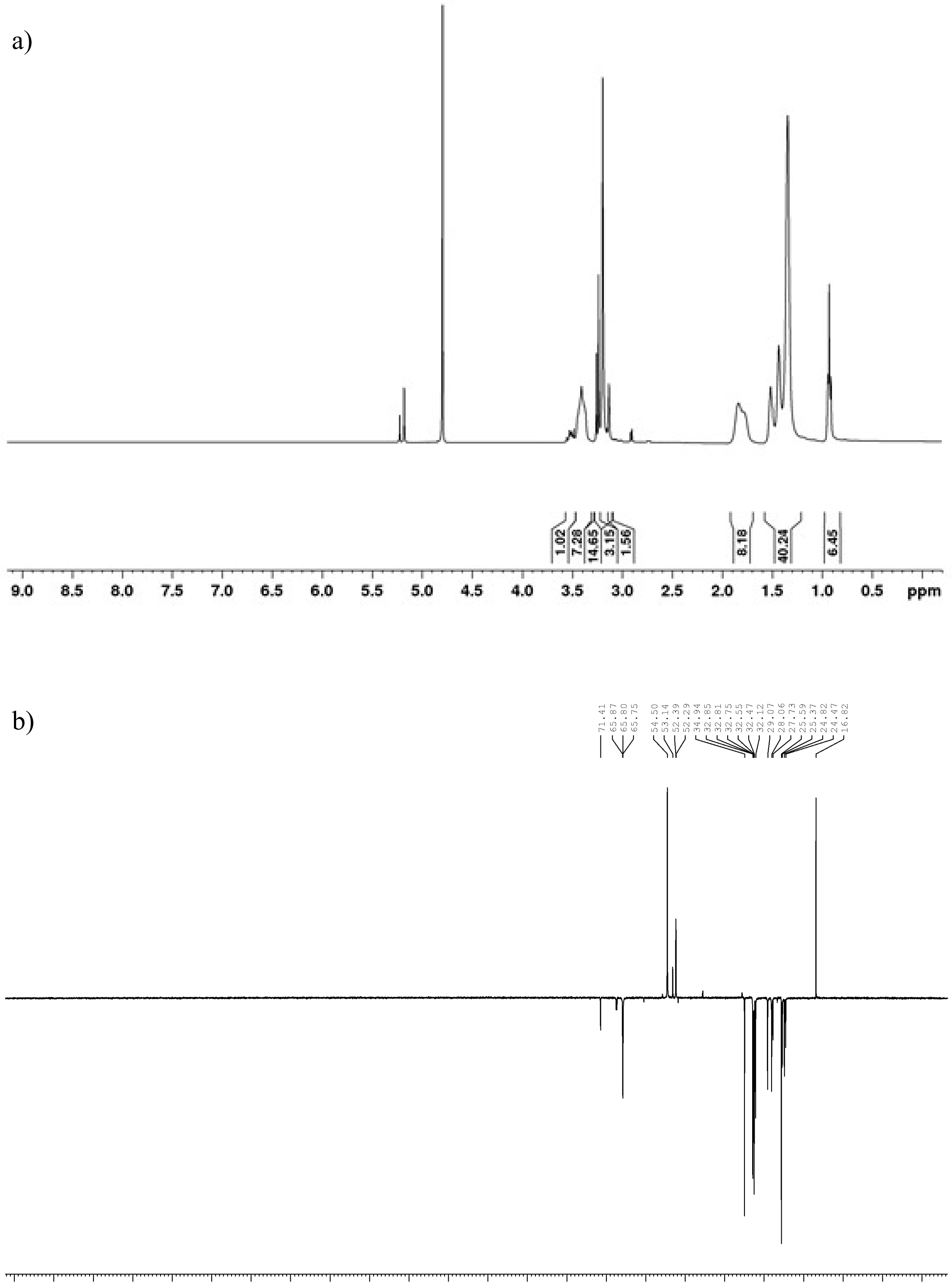

Figure S5. a) ^{1}H and b) ^{13}C NMR spectra of the synthesized *N*,*N′*-didodecyl-*N*,*N*,*N′*,*N′*-tetramethylhexane-1,6-diammonium dibromide (12-6-12).

a) ^{1}H NMR (D_2O), 0.93, t, 6H, 2CH_3 from

2$NCH_2CH_2CH_2CH_2CH_2CH_2CH_2CH_2CH_2CH_2CH_2CH_3$,

1.24-1.56, m, 40H, 2$NCH_2CH_2CH_2CH_2CH_2CH_2CH_2CH_2CH_2CH_2CH_2CH_3$,

$NCH_2CH_2CH_2CH_2CH_2CH_2N$,

1.71-1.92, m, 8H, 2$NCH_2CH_2CH_2CH_2CH_2CH_2CH_2CH_2CH_2CH_2CH_2CH_3$,

$NCH_2CH_2CH_2CH_2CH_2CH_2N$,

3.20, s, 12H, 2$N(CH_3)_2$,

3.31-3.51, m, 8H, 2$NCH_2CH_2CH_2CH_2CH_2CH_2CH_2CH_2CH_2CH_2CH_2CH_3$,

$NCH_2CH_2CH_2CH_2CH_2CH_2N$,

b) ^{13}C NMR (D_2O), 16.82 ($NCH_2CH_2CH_2CH_2CH_2CH_2CH_2CH_2CH_2CH_2CH_2CH_3$),

24.47, 25.59, 28.06, 29.07, 32.12, 32.47, 32.55, 32.75, 32.81, 32.85, 34.94

($NCH_2CH_2CH_2CH_2CH_2CH_2N$), ($NCH_2CH_2CH_2CH_2CH_2CH_2CH_2CH_2CH_2CH_2CH_2CH_3$)

54.50 (NCH_3),

65.75 ($NCH_2CH_2CH_2CH_2CH_2CH_2N$), ($NCH_2CH_2CH_2CH_2CH_2CH_2CH_2CH_2CH_2CH_2CH_2CH_3$).

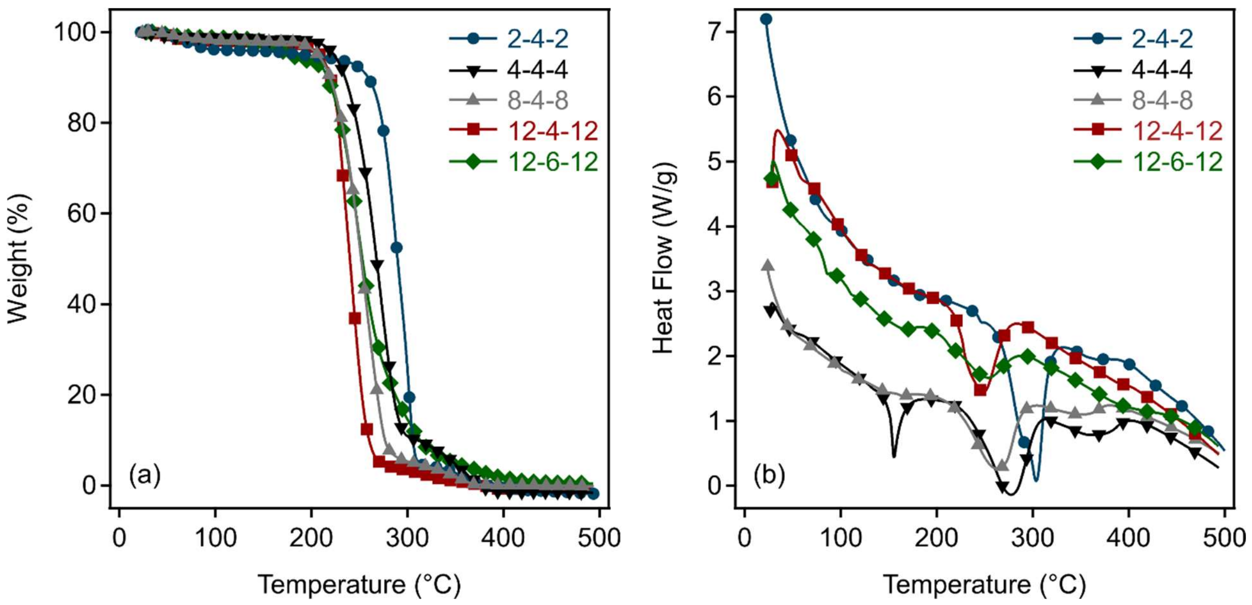

Figure S6. TG (a) and DSC (b) curves recorded during thermal decomposition for the gemini surfactants investigated.

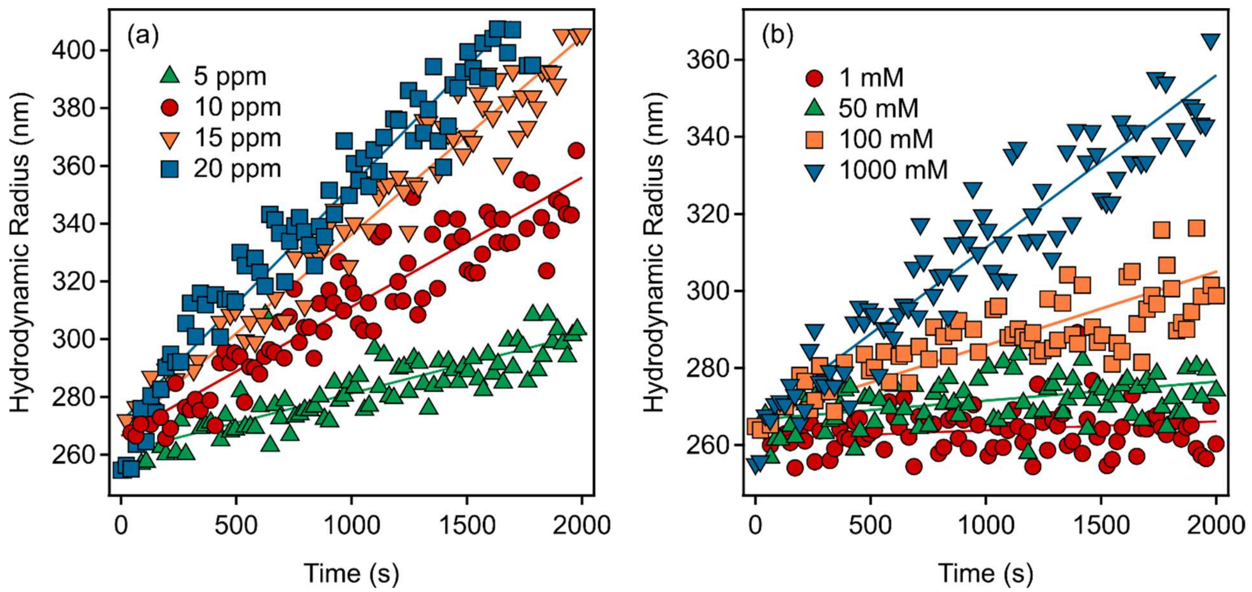

Figure S7. Time-resolved DLS measurements at different initial SL particle concentrations at 1 M ionic strength (a) and at 10 mg/L SL concentration and different KCl concentrations (b) at pH 4. The solid lines are linear fits used to calculate the absolute aggregation rate in eq 3.

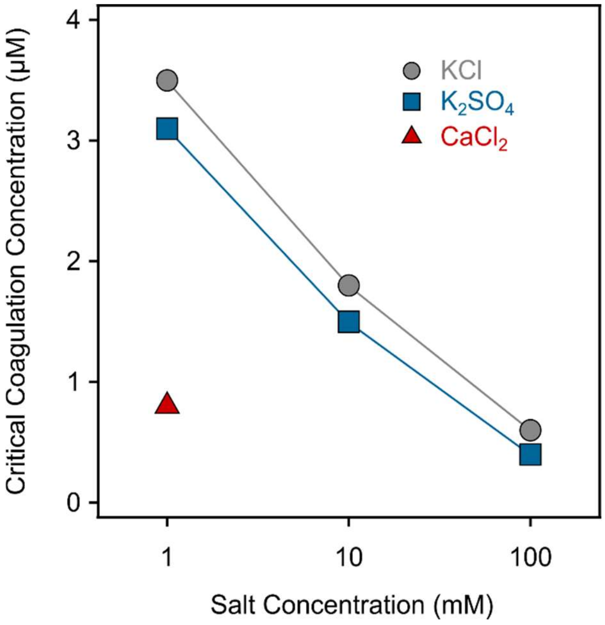

Figure S8. Critical coagulation concentration values of SL particles as a function of salt concentration in the presence of 12-4-12 at pH 4. The solid lines are just to guide the eyes.

Table S1. Provenance, purity, structure, and molar mass of the chemicals used for the synthesis of the gemini surfactants.

Chemical Name	Structure	Source	Molar mass g/mol	CAS number	Mass fraction purity
1-Bromoethane	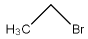	Sigma-Aldrich	108.97	74-96-4	98%
1-Bromobutane	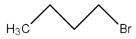	Sigma-Aldrich	137.02	109-65-9	99%
1-Bromooctane	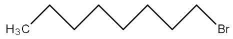	Sigma-Aldrich	193.12	111-83-1	99%
1-Bromododecane	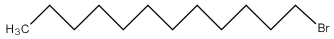	Acros Organics	249.23	143-15-7	98%
N,*N*,*N*',*N*'-tetramethyl-1,4-butanediamine	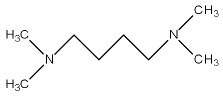	Sigma-Aldrich	144.26	111-51-3	98%
N,*N*,*N*',*N*'-tetramethyl-1,6-hexsanediamine	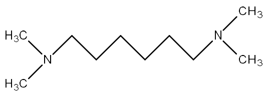	Sigma-Aldrich	172.31	111-18-2	99%
Acetonitrile		Sigma-Aldrich	41.05	75-05-8	≥99.5%
Ethyl acetate	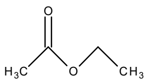	Sigma-Aldrich	88.11	141-78-6	≥99.5%

Table S2. Characteristic charging and aggregation data for SL particles in the presence of inorganic electrolytes.

Salt	σ (mC/m^2)[a]	CCIS (mM)[b]	CCC (mM)[c]
KCl	−15	90	90
K_2SO_4	−25	150	50
$CaCl_2$	−8	30	10

[a]Charge density determined with eq 2. [b]Critical coagulation ionic strength determined based on data presented in Figure 5. [c]Critical coagulation concentration was calculated from CCIS using eq 6.

Table S3. Isoelectric point and critical coagulation concentration values of SL particles in the presence of 12-4-12 surfactant and different background electrolytes.

	Ionic strength (mM)	IEP (μM)[a]	CCC (μM)[b]
KCl	1	5.00	3.50
	10	2.70	1.80
	100	1.06	0.60
K_2SO_4	3	3.80	3.10
	30	1.60	1.30
	300	0.80	0.40
$CaCl_2$	3	2.50	0.80
	30	0.50	–[c]
	300	0.30	–[c]

[a]Concentration of 12-4-12 needed to reach the IEP with sulfate latex particles. [b]12-4-12 concentration required to attain the CCC of SL particles. [c]CCC could not be determined since the stability ratio was already close to unity at low 12-4-12 doses.
